# Regulation of sub-compartmental targeting and folding properties of the Prion-like protein Shadoo

**DOI:** 10.1038/s41598-017-03969-2

**Published:** 2017-06-16

**Authors:** Anna Pepe, Rosario Avolio, Danilo Swann Matassa, Franca Esposito, Lucio Nitsch, Chiara Zurzolo, Simona Paladino, Daniela Sarnataro

**Affiliations:** 1 Department of Molecular Medicine and Medical Biotechnology, University of Naples “Federico II”, Via Pansini 5-80131 Naples, Italy; 2 Ceinge-Biotecnologie avanzate, s.c.a r.l., Via G. Salvatore, 486-80145 Naples, Italy; 3 Unité de Trafic Membranaire et Pathogenese, Institut Pasteur, 25-28 Rue du Docteur Roux, 75724 Paris, CEDEX 15 France

## Abstract

Shadoo (Sho), a member of prion protein family, has been shown to prevent embryonic lethality in *Prnp* ^0/0^ mice and to be reduced in the brains of rodents with terminal prion diseases. Sho can also affect PrP structural dynamics and can increase the prion conversion into its misfolded isoform (PrP^Sc^), which is amyloidogenic and strictly related to expression, intracellular localization and association of PrP^C^ to lipid rafts. We reasoned that if Sho possesses a natural tendency to convert to amyloid-like forms *in vitro*, it should be able to exhibit “prion-like” properties, such as PK-resistance and aggregation state, also in live cells. We tested this hypothesis, by different approaches in neuronal cells, finding that Sho shows folding properties partially dependent on lipid rafts integrity whose alteration, as well as proteasomal block, regulated generation of intermediate Sho isoforms and exacerbated its misfolding. Moreover, a 18 kDa isoform of Sho, likely bearing the signal peptide, was targeted to mitochondria by interacting with the molecular chaperone TRAP1 which, in turn controlled Sho dual targeting to ER or mitochondria. Our studies contribute to understand the role of molecular chaperones and of PrP-related folding intermediates in “prion-like” conversion.

## Introduction

Prion diseases are a group of rare, infectious, sporadic or inherited neurodegenerative disorders in mammals characterized by a primary pathogenic event consisting of a conversion of the cellular prion protein (PrP^C^) into a disease-associated misfolded isoform PrP^Sc^ ^1, 2^. It is likely that in the inherited forms of the disease the conversion occurs as a result of a dominant mutation in the PrP gene, which promotes conformational transition into a β-sheets enriched structure. Despite intensive research over years, the exact mechanism by which the conversion occurs is poorly understood^3^, as are the exact function of PrP^C^ in the cells and the phenotypic impacts of conformational variations and protein partners of PrP^C^. Furthermore, in the last years it has emerged that prion protein family includes two other members in addition to PrP^C^, the so called Doppel and Shadoo (Sho), this latter being discovered *in silico* ^4^ and mainly expressed in the central nervous system (CNS)^5^. In linear domain alignments, Sho resembles both the central and N-terminal regions of PrP^C^ and the conformational and assembly properties of PrP^C^ hydrophobic domain^6^. Besides structural similarities, several other analogies have been identified between PrP and Sho: their highest levels in the CNS^5, 7^, endoproteolitic fragments adjacent to glycophosphatidyl-inositol (GPI) anchor^6, 8, 9^, and their rescue effects on toxic phenotype exerted, in certain conditions, by the third member of the family, Doppel^5^. In addition, several data from PRNP-knockout mice suggest that PrP^C^ and Sho have overlapping functions^10, 11^ but this issue is far from being clarified.

The subcellular localization of both proteins is predicted to be the same, as both possess a GPI anchor and N-glycosylation sites that are suggestive of their entrance to the secretory pathway^4, 7^. The presence of a GPI-attachment signal and N-glycosylation were experimentally demonstrated for the endogenous protein in brain homogenates as well as for recombinant Sho^5, 12^.

We have previously shown that alteration of detergent-resistant membranes (DRMs), also called lipid rafts, by cholesterol depletion, leads to PrP^C^ misfolding in the endoplasmic reticulum (ER)^13, 14^ and that the ER-associated degradation pathway (ERAD) is involved in the processing of the non-translocated form of PrP^C^ in epithelial cells^15^.

Here, we have characterized the subcellular localization of endogenously expressed Sho in neuronal (GT1 and SH-SY5Y) and non neuronal (HeLa) cells, its association with DRMs and their role, which has never been tested before, in its folding.

Our study shows that, while the unglycosylated and mature Sho isoforms are partially present in the ER, exclusively a 18 kDa isoform of Sho, possibly bearing its signal peptide, localizes to mitochondria. Interestingly, we found that Sho interacts with the mitochondrial chaperone TRAP1, whose expression levels control dual ER/Mitochondrial targeting of Sho, thus regulating the correct Sho localization at the interface between the two compartments.

Furthermore, for the first time we also show that Sho possesses “prion-like” characteristics, such as PK-resistance and aggregation properties, already under normal growth conditions and associates with ER chaperones, indicating that it tends to misfold. Interestingly, as for PrP^C^, cholesterol depletion leads to an accumulation of the unglycosylated isoform and exacerbates the “prion-like” features of Sho. Finally, we demonstrated a proteasome control in the degradation of this latter isoform.

## Methods

### Reagents and antibodies

Cell culture reagents and Mitotracker green were purchased from Life Technologies Laboratories (Grand Island, NY). Anti-Sho SPRN R-12 antibody sc-136909^16^ (mapping within an internal region of SPRN of mouse origin), anti-TRAP1 Ab (73604), Bip/Grp78 (sc-1051), F1ATPase-ATP5B subunit (sc-58619), TOM20 (sc-17764) and anti-GAPDH (sc-69778) were from Santa Cruz Biotechnology. Monoclonal SAF32 antibody against PrP^C^ was from Cayman Chemical (USA), Sha31 anti-PrP monoclonal Ab (A03213) was from SPA-Bio. The antibody against Calreticulin (CRT), Golgin, PDI and KDEL were from StressGen Biotechnologies Corp. (Victoria, British Columbia, Canada). Anti-Calnexin (CNX) antibody was from ThermoFisher Scientific (MA5-15385). ALLN was from Calbiochem (La Jolla, CA). AlexaFluor-488- and 546- and cy5- conjugated secondary antibodies were from Invitrogen (Molecular Probes). DRAQ5 and DAPI dyes were purchased from Cell Signal Technology. Protein A-Sepharose was from Pharmacia Diagnostics AB (Uppsala, Sweden). Methyl-β-cyclodextrin (βCD), and all other reagents were obtained from Sigma Chemical Co. (St. Louis, MO).

### Cell culture and drugs treatment

Chimeric cDNA encoding the different eGFP-tagged TRAP1 was amplified by PCR from already established eGFP-containing-plasmid libraries^17^. Resulting chimeric cDNA was cloned into pCDNA5/FRT/TO (Life Technologies). HeLa cell line expressing TRAP1-GFP was established as described in the manufacturer’s protocols (Flp-In TRex, Life Technologies). Parental HeLa Flp-In TRex and HeLa cell line expressing unfused GFP were a kind gift from Matthias Hentze.

GT1 and HeLa cells were grown in Dulbecco’s modified Eagle’s medium (DMEM), with 4500 mg/glucose/L, 110 mg sodium pyruvate and L-glutamine (SIGMA D6429), supplemented with 10% fetal bovine serum. SH-SY5Y were grown in RPMI-1640 (Euroclone), with 4500 mg/glucose/L, 110 mg sodium pyruvate and L-glutamine, supplemented with 10% fetal bovine serum. Cells were cultured at 37 °C under 5% CO2. Proteasomal block was performed with 150 μM ALLN for 7 hours in complete medium. Methyl-β-cyclodextrin (βCD) treatment was carried out as described elsewhere^18^. Briefly, GT1 or SH-SY5Y cells were plated on dishes and cholesterol depleted by βCD (10 mM) that was added to the medium containing 20 mM HEPES, pH 7.5, and 0.2% bovine albumin for 1 h at 37 °C.

#### Cholesterol determination by colorimetric assay

GT1 and SH-SY5Y cells in the presence or absence of βCD were washed twice with PBS, lysed with appropriate lysis buffer and Infinity Cholesterol Reagent (Sigma Chemical Co. St Louis, MO, USA, code 401–25 P) was added to the lysates in the ratio 1:10 for 5 min at 37 °C (according to the suggested Sigma protocol number 401). The samples were then read at the spectrophotometer at 550 nm wavelength.

### PNGase F digestion

Peptide N-glycosidase (PNGaseF, Roche11365177001) digestion was performed on lysated samples. The cells were lysed in Buffer 1 (25 mM Tris Hcl pH 7,5, 150 mM NaCl, 5 mM EDTA, 1% TX-110) on ice for 20 min; nuclei were pelleted at 3,000 rpm for 5 min, supernatant was boiled for 5 min at 95 °C, and adjusted to 20 mM Tris pH 7.5, 2 mM EDTA, 0.1% SDS, 1% NP40, 1% (v/v) 2-mercaptoethanol then incubated with PNGaseF (5 units/sample) for 16 h at 37 °C. The samples were analyzed by SDS-PAGE and Western blotting.

### Confocal microscopy

GT1 and SH-SY5Y cells were grown for 3 days on coverslips, washed with PBS, fixed and processed for indirect immunofluorescence (the cells were permeabilized with methanol/acetone 1:1 at −20 °C for 5 min) using anti-Sho SPRN-R12 pAb. Pearson Correlation Coefficient (PCC) was employed to quantify colocalization^19^. PCC was calculated in regions of Sho and reference protein co-presence. In brief, the Otsu algorithm was applied to segment Sho and KDEL or Mitotracker green images, in order to define co-localization regions of the reference proteins. The PCC was then calculated in the defined regions for the images of interest.

Digitonin Permeabilization. SH-SY5Y cells grown for 3 days on coverslips were washed twice with a specific buffer (20 mM Hepes-KOH, pH 7.2, 110 mM Potassium acetate, 2 mM Magnesium acetate) and then incubated on ice for 5 min with digitonin (20 μg/ml) in the buffer composed as described above^20^. After washing, coverslips were fixed in 2% paraformaldehyde and, where indicated, permeabilized with 0.075% saponin, processed for indirect immunofluorescence, and analyzed by confocal microscopy.

Immunofluorescences were analyzed by the confocal microscope Zeiss META 510 equipped with an oil immersion 63 × 1.4 NA Plan Apochromat objective, and a pinhole size of one airy unit. We collected twelve-bit confocal image stacks of 10–15 slices at 0.5 µm Z-step sizes from dual- or triple-labeled cells using the following settings: green channel for detecting Alexa-488, excitation 488 nm Argon laser, emission bandpass filter 505–550 nm; red channel for detecting Alexa-546, excitation 543 nm Helium/Neon laser, emission bandpass filter 560–700 nm (by using the meta monochromator); blue channel for detecting DAPI, excitation 405 nm blue diode laser, and emission bandpass 420–480 nm; blue channel for detecting DRAQ5 or cy5-conjugated secondary antibodies, excitation 633 nm Helium/Neon laser.

Measurements of fluorescence intensity were taken on a minimum of three confocal stacks per condition, from a single experiment (~80 cells), using LSM 510 Zeiss software. The background values raised by fluorescent secondary antibodies alone, were subtracted from all samples.

Colocalization between Sho and Mitotracker green was determined in at least 52 cells from three different experiments under control or TRAP1 silenced or induced cells. The analysis was performed by LSM 510 software and the number of colocalizing pixels was normalized for the total number of pixels from an extracted region of interest in the confocal image.

### Biotinylation assay

GT1 cells grown on dishes were cooled on ice and biotinylated with NHS-LC-Biotin at 4 °C. Cells were lysed for 20 min using Buffer 1 (25 mM Tris-HCl pH 7.5, 150 mM NaCl, 5 mM EDTA, 1% TX-100). Biotinylated cell surface proteins were immunoprecipitated with streptavidin beads (40 μl/sample, Pierce n. 20349). Sho was specifically immunorevealed with the SPRN-R12 Ab.

### QproteomeTM Mitochondria Isolation

Qproteome Mitochondria Isolation kit from Qiagen (37612) was used to purify mitochondria from 6 × 10^6^ SH-SY5Y or HeLa cells. The cell suspension was centrifuged at 500 × *g* for 10 min at 4 °C. Supernatant was removed and the pellet was washed using 1 ml of 0.9% NaCl solution for two times. After washing, cells were suspended with 1.5 ml of Lysis Buffer (protease inhibitor solution was added) which selectively disrupts the plasma membrane without solubilizing it, resulting in the isolation of cytosolic proteins. Plasma membrane and compartmentalized organelles, such as nuclei, mitochondria, and the endoplasmic reticulum (ER), remained intact and were pelleted by centrifugation at 1000 × *g* for 10 min at 4 °C. The resulting pellet was resuspended in 1.5 ml of Disruption Buffer, repeatedly passed through a narrow-gauge needle (to ensure complete cell disruption), and centrifuged at 1000 × *g* for 10 min at 4 °C.

The pellet containing nuclei, cell debris, and unbroken cells was discarded and the supernatants were centrifuged at 6000 × *g* for 10 min at 4 °C to isolate mitochondria. The pellet contains mitochondria. The supernatant represents the microsomal fraction. The mitochondrial pellet was washed with 1 ml Mitochondria Storage Buffer and then Centrifuged at 6000 × *g* for 20 min at 4 °C. Then the mitochondrial pellet was resuspended with 150 μl of Mitochondria Storage Buffer.

Each isolated fraction was quantified with Breadford assay with Bio-Rad Protein Assay Dye Reagent, diluited 1:5 in water. Equal amounts of protein of each fraction (InPut-Cyt-ER-Mito) were boiled with SDS-sample buffer 2X, loaded on 14% polyacrylamide gel and reveled by western blotting with SPRN-R12 antibody to reveal Sho. PVDF membranes were then probed with anti-GAPDH, anti-F1ATPase and anti-BiP Abs, as cytosol- mitochondria- and ER-markers, respectively.

### Assays for DRM-association

#### TX-100 extraction

Cells grown in 60-mm dishes were washed twince with PBS containing 1 mM CaCl_2_ and 1 mM MgCl_2_ (PBS C/M) and then lysed for 20 min on ice in 1 ml Extraction Buffer (25 mM Hepes pH 7.5, 150 mM NaCl, 1% TX-100). Lysates were collected and centrifuged at 14000 r.p.m. for 2 min at 4 °C. Supernatants, representing the soluble material, were removed and 1% SDS was added; the pellets were then solubilized in 100 μl of Solubilization buffer (50 mM Tris pH 8.8, 5 mM EDTA, 1% SDS). DNA was sheared through a 22-g needle. The pellets were solved, boiled 3 min and 900 μl of Extraction buffer was added. Proteins were TCA precipitated from the soluble and insoluble materials and Sho was revealed by Western blotting with R-12 antibody.

#### Sucrose density gradients

Cells were grown to confluence in 150-mm dishes, washed in PBS C/M and lysed for 20 min in 1% TNE/TX-100 on ice^13, 21^. Lysates were scraped from dishes and sheared though a 22-g needle and then centrifuged at 14.000 r.p.m. 10 min at 4 °C. Supernatants were placed at the bottom of centrifuge tube, brought to 40% sucrose. A discontinuous sucrose gradient (5–35% TNE) was layered on the top of the lysates and the samples were centrifuged at 39.000 r.p.m. for 18 h in an ultracentrifuge (model SW41 Beckman Institute, Fullerton, CA, USA). One-milliliter fractions (12 fractions in total) were harvested from the top of the gradient. Specifically, starting from the top of the gradient the fractions 4–7 (representing DRMs) and 8–12 (non-DRMs) were collected and loaded on gel. After transfer on PVDF by Western blot, Sho, PrP^C^ and Flotillin-2 were revealed by specific antibodies and ECL.

### Assays for “prion-like” properties

#### Triton/Doc insolubility

Cells were lysed in Triton/Doc buffer (0.5% Triton X-100, 0.5 Na Deoxicolate, 150 mM NaCl and 100 mM Tris, pH 7.5) for 20 min and cleared lysates were centrifuged at 265000 × *g* for 40 min in a TLA 100.3 rotor of Beckman Optima TL ultracentrifuge. Sho was recovered in the supernatants and pelleted by TCA precipitation. It has been shown that in these conditions only PrP^Sc^ but not PrP^C^ from brain extracts and cell culture lysates (from CHO, NIH 3T3 or neuroblastoma cells) will sediment^22, 23^.

#### Proteinase-K digestion

To measure proteinase K-resistance, lysates were digested with proteinase- K (3.3 μg/ml or 20 μg/ml, as indicated) for 2 and 10 min at 37 °C; the proteins were TCA precipitated and then analyzed for Sho by immunoblotting with the specific antibody. The conditions used for proteinase digestion are identical to those previously published^14, 22, 23^.

### Immunoprecipitation of Molecular Chaperones

To immunoprecipitate Calreticulin (CRT) the cells were grown in 100 mm dishes, washed three times with cold PBS and lysed in JS buffer (1% TX-100, 150 mM NaCl, 1% Glycerol, 50 mM HEPES, pH 7.5, 1.5 mM MgCl2, 5 mM EGTA) with protease inhibitor coktail, for 20 min on ice, scraped and put in microfuge tubes. The lysates were then precleared with protein A-Sepharose beads (5 mg/sample) for 30 min and incubated overnight at 4 °C with anti-CRT Ab. The pellets were washed twice with cold lysis buffer and three times with PBS. The samples were then boiled with SDS-sample buffer^14^. TRAP-1 immunoprecipitation was carried out on 1,5 mg of total cell extracts. Cells were lysed in cold lysis Buffer (20 mM Tris pH 7.5, 60 mM KCl, 15 mM NaCl, 2 mM EDTA, 1% (vol/vol) Triton X-100, 1 mM PMSF, 2 mg/ml aprotinin, 2 mg/ml Leupeptin). Protein concentration was quantified using the Bio-Rad protein assay kit (Bio-Rad Laboratories). Lysates were incubated O/N with 50 μl (1 mg/ml) protein Protein A/G magnetic beads (Bio-Rad) and anti-TRAP-1 Ab (1 μg/ml). Negative control experiments were performed by adding magnetic beads and IgM to the cleared lysates. Then, the Co-IP was washed with PBS-T (PBS + 0.1% Tween 20), the beads were magnetized and 1/10 supernatants were loaded for reference. Pellets were boiled with SDS sample buffer 2X at 95 °C. The samples were loaded on 14% polyacrylamide gels and revealed by Western blotting with anti-Sho, anti-CRT or TRAP-1 Abs.

### Statistical analysis

Statistical significance of samples against untreated cells was determined by One-way analysis of Variance (ANOVA), followed by the Dunnett’s test. Each value represents the mean ± S.D. of at least three independent experiments performed in triplicate (*P < 0.05).

## Results

### Endogenous Sho and PrP^C^ expression and localization in neuronal cells

**Figure 1:**
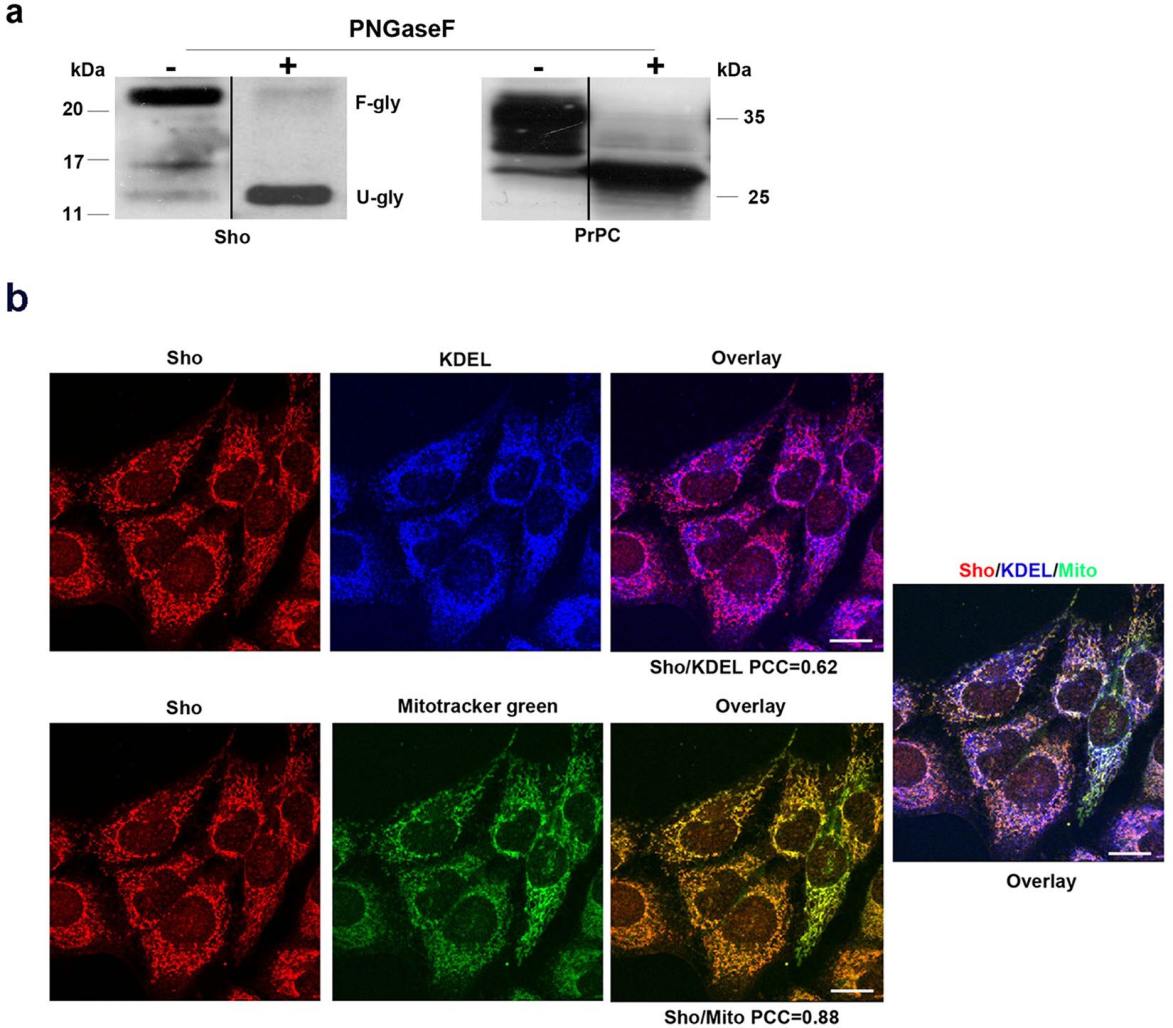
Sho is localized in the ER and mitochondria of neuronal cells. (**a**) GT1 cells were grown on dishes and Peptide N-glycosidase (PNGaseF) digestion (5 units/sample) was performed on 1 mg of cell lysates. Treated (+) or not (−) samples were loaded on gels and then analyzed by SDS-PAGE and Western blotting with anti-Sho and anti-PrP SAF32 Abs. F-gly: fully glycosylated, U-gly, unglycosylated. The band of Sho at ~16 kDa is sensitive to PNGaseF and could be an intermediate glycosylation product. (**b**) GT1 cells, grown on coverslips, were fixed and incubated with anti-Sho SPRN-R12 and anti-KDEL primary antibodies followed by secondary antibodies conjugated to Alexa-fluor546 (red) and Cy5 (blue) to localize Sho and KDEL, respectively. To localize mitochondria, Mitotracker green (1 mM) was added to live cells into cell culture medium 15 minutes before fixation. The overlay of red/green and blue channels is shown on the right panel. PCC is an average value (N = 80) and based on at least four independent experiments. *P* < 0.03. Scale bars: 10 μm.

The endogenous Sho, from both mouse neuronal GT1 and human SH-SY5Y cell lysates, migrated on SDS-PAGE as different bands corresponding, respectively, to the glycosylated (~22 kDa) and unglycosylated (~14 kDa) form, as shown before^5^ (Fig. 1a and Supplementary Fig. S1). By using PNGaseF de-glycosylation enzyme, we found that Sho was complex-glycosylated in both cell lines and that the band at 16 kDa, which is sensitive to PNGaseF, is an intermediate glycosylation isoform of Sho.

PrP^C^ was carried as control of the procedure. As already observed^24^, PrP^C^ was typically glycosylated, showing different bands ranging from ~27 to ~37 kDa; the unglycosylated form which migrates at ~27 kDa, the intermediate monoglycosylated form, migrating at 28–30 kDa; and the highly glycosylated forms as bands spanning 33–37 kDa.

Based on a previous report (where different transfected Sho constructs were analyzed in both SH-SY5Y and N2a cells) showing that the ER signal peptide of Sho can mediate targeting to mitochondria^25^, we decided to explore the subcellular localization of endogenous Sho, using indirect immunofluorescence microscopy followed by confocal analysis. Strikingly, we found that Sho colocalized both with the ER marker KDEL (PCC = 0.62) and mitochondrial marker Mitotracker green (PCC = 0.88, N = 80) (Fig. 1b). In addition, to strengthen these findings, we performed an immunofluorescence assay under conditions where the detergent digitonin was used to only limited and selectively permeabilize plasma membrane (see also ref. 20). The permeabilization with saponin after digitonin would allow to permeabilize the membrane organelles and to reach the organelle lumen to verify the presence or absence of a specific molecule inside. Employing this approach we expected that a protein with the epitopes for its Ab exposed in the lumen of organelles, should not have been labelled under digitonin alone; while a membrane protein with exposed cytosolic epitopes should have been marked. Furthermore, following saponin permeabilization we should have been able to reach and label lumen protein epitopes. To this aim we selected different markers of the mitochondria, F1ATPase and TOM20 (as inner and outer mitochondrial membrane, respectively) and of ER, CNX and PDI (as membrane and lumen ER marker), in a double IF assay with R12 anti-Sho Ab (which recognizes an internal N-terminal region of the protein, see new Supplementary Figs S2 and S3). As expected, when we permeabilized with digitonin only, both mitochondrial F1ATPase and ER lumenal PDI, were not detectable. Under these conditions, using digitonin alone we found colocalization between Sho and CNX (PCC = 0.66) but not with TOM20 (PCC = 0.12), suggesting localization of Sho in closely apposition to the cytoplasmic surface of ER compartment, possibly corresponding with the pool of Sho molecules which did not translocate in the secretory pathway. After saponin permeabilization (allowing the access to the lumen of organelles), we calculated a good degree of colocalization between Sho and F1ATPase (PCC = 0.82), as well as with PDI (PCC = 0.72), but again not with TOM20 (PCC = 0.13). All together these data reinforce our observation that Sho localizes inside these organelles without excluding the possibility it could associate to the outside surface of ER.

Furthermore, in agreement with previous findings^25^, Sho was also present on the cell surface but, in contrast to previous observations^25^, it did not localize in the Golgi apparatus (see Supplementary Fig. S4). This apparent discrepancy could be ascribed to the fact that endogenous Sho protein (not the transfected one) was analysed in this study, and that its transit through the Golgi complex is very rapid; thus, the possibility to find Sho accumulating in this secretory compartment is likely lower compared to transfected conditions.

### ER/Mitochondrial localization of Sho and TRAP1 chaperoning

**Figure 2:**
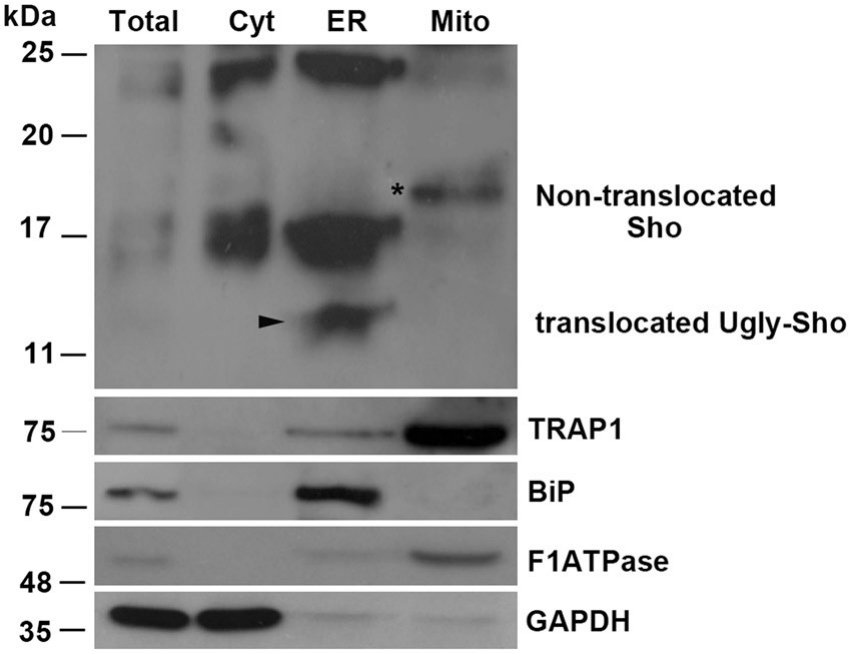
A 18 kDa isoform of Sho is targeted to mitochondria. GT1 cells were processed for mitochondria fractionation assay (see methods for procedure). Total represents the InPut; Cyt: cytosol; ER: microsomal fraction; Mito: mitochondrial fraction. Western blotting with anti- BiP (ER marker), anti-F1ATPase (mitochondrial marker), anti-GAPDH (cytosolic marker) and anti-TRAP1 antibodies were performed as control of the procedure. Note the presence of the 18 kDa Sho isoform only in the mitochondrial fraction (asterisk). At least four independent experiments were analysed.

Because mitochondrial localization for a secretory protein is not likely expected, we decided to perform western blot analysis of purified mitochondrial fraction from GT1 cells (Fig. 2), and other neuronal and non neuronal cells (see also below). We found that specifically an isoform of Sho with an electrophoretic motility around ~18 kDa (asterisk) co-purified with mitochondria. Reasonably, and in agreement with previous results^25^, this latter form could likely correspond to the non-translocated Sho, which should retain the N-terminal ER-signal peptide.

Furthermore, the fractionation assay of subcellular compartments allowed us to show that three isoforms of Sho enriched the ER: the fully glycosylated (22 kDa), the partially glycosylated translocated Sho (~16 kDa) and the unglycosylated ~14 kDa isoform (Fig. 2).

The recently proposed role for the mitochondrial chaperone TRAP1 (Heat shock protein 75 kDa) both in protein quality control for mistargeted/misfolded mitochondria-destined proteins^26^ and in crosstalk between mitochondria and other subcellular compartments^27^, prompted us to check for Sho/TRAP1 interaction. Interestingly, in agreement with previous description by Amoroso *et al*.^26^, TRAP1 was recovered from mitochondrial fraction but a fair amount was also revealed in the ER fraction (see Fig. 2).

**Figure 3:**
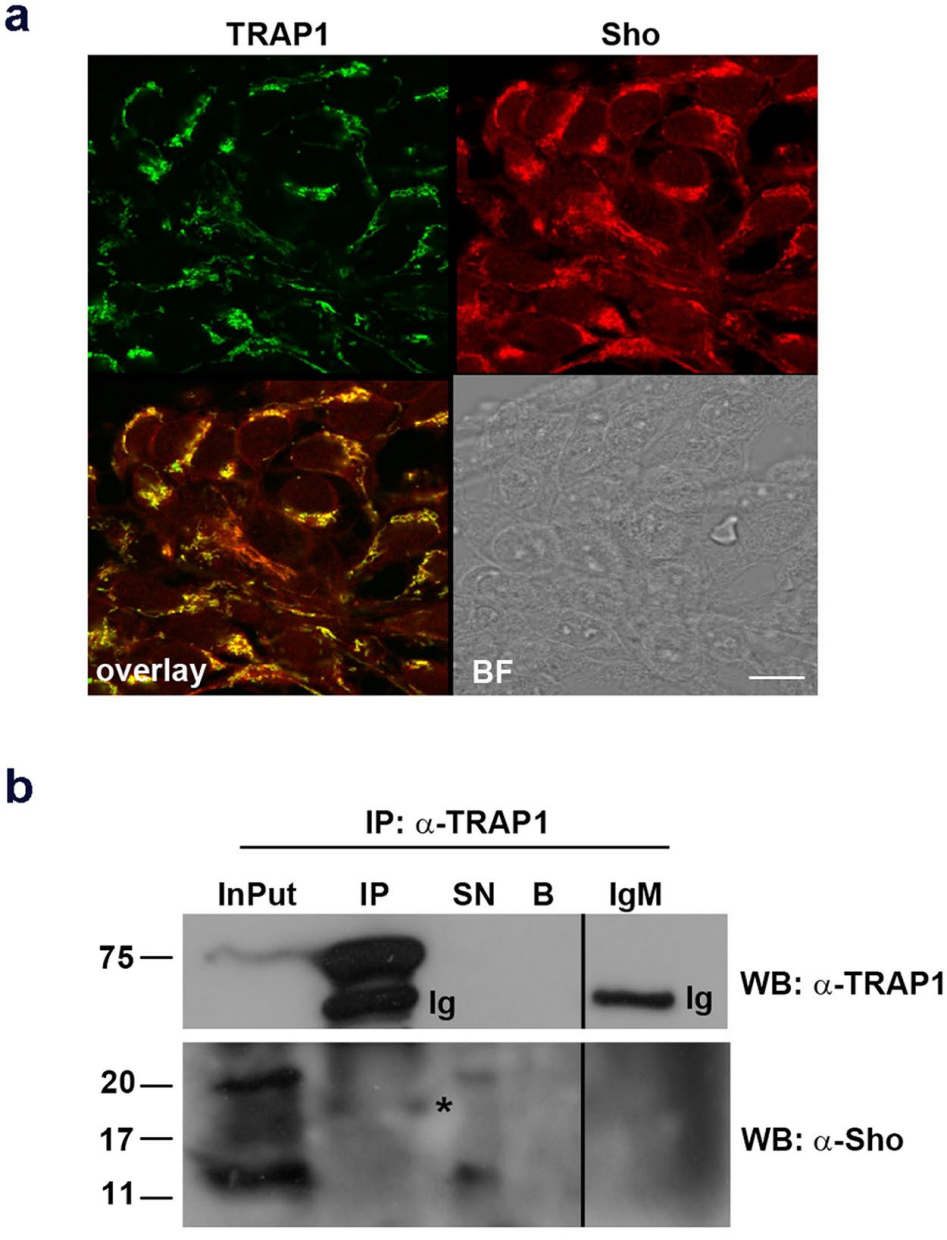
Sho colocalizes with TRAP1 and its 18 kDa isoform co-immunoprecipitates with TRAP1. (**a**) SH-SY5Y cells were grown on coverslips and processed for double immunofluorescence analysis probing with anti-TRAP1 and anti-Sho SPRN-R12 Abs followed by secondary Abs conjugated to Alexa-fluor488 and Alexa-fluor546, to reveal TRAP1 (green) and Sho (red) respectively. Brightfield (BF) and overlay images are shown. Colocalization between TRAP1 and Sho was calculated from three independent experiments as described in methods section. PCC = 0.78 (N = 80 cells). *P* < 0.05. Scale bars: 10 μm. (**b**) SH-SY5Y cells were lysed in Buffer 1 and TRAP1 was immunoprecipitated (IP) using the monoclonal anti-TRAP1 Ab (73604 Santa Cruz). The immunoprecipitated material was then immunolabeled with the anti-Sho antibody SPRN-R12. (Ig) indicates the immunoglobulins and (*) indicates 18 kDa Sho. To confirm the occurrence of the immunoprecipitation, the membranes were stripped and probed with the anti-TRAP1 antibody. InPut: loading control (50 μg of total lysate); IP: immunoprecipitate; SN: 1/10 of the supernatant; B: magnetic beads alone.

Thus, in order to investigate whether Sho and TRAP1 entertained physical interaction we employed the human neuronal SH-SY5Y cells. We first subjected the cells to double immunofluorescence assay with anti-Sho SPRN-R12 Ab and with anti-TRAP1 Ab (Fig. 3a) finding a good degree of colocalization between TRAP1 and Sho; then in parallel, total cell lysates were processed for co-immunoprecipitation (Co-IP) assays (Fig. 3b). We first immunoprecipitated TRAP1 from lysates and then immuno-identified Sho in the precipitate by Western blotting with SPRN-R12 antibody. As deduced by migration of standard molecular weights and as shown in the Fig. 3b, the ~18 kDa isoform of Sho (asterisk) could be immunoprecipitated along with TRAP1, whereas the other isoforms remained in the supernatant (SN) of the immunoprecipitate. Importantly, to confirm the specificity of the co-immunoprecipitation, we used as control non-specific immunoglobulins (IgM) in the precipitation step or magnetic beads alone.

### TRAP1 expression regulates mitochondrial localization of Sho

**Figure 4:**
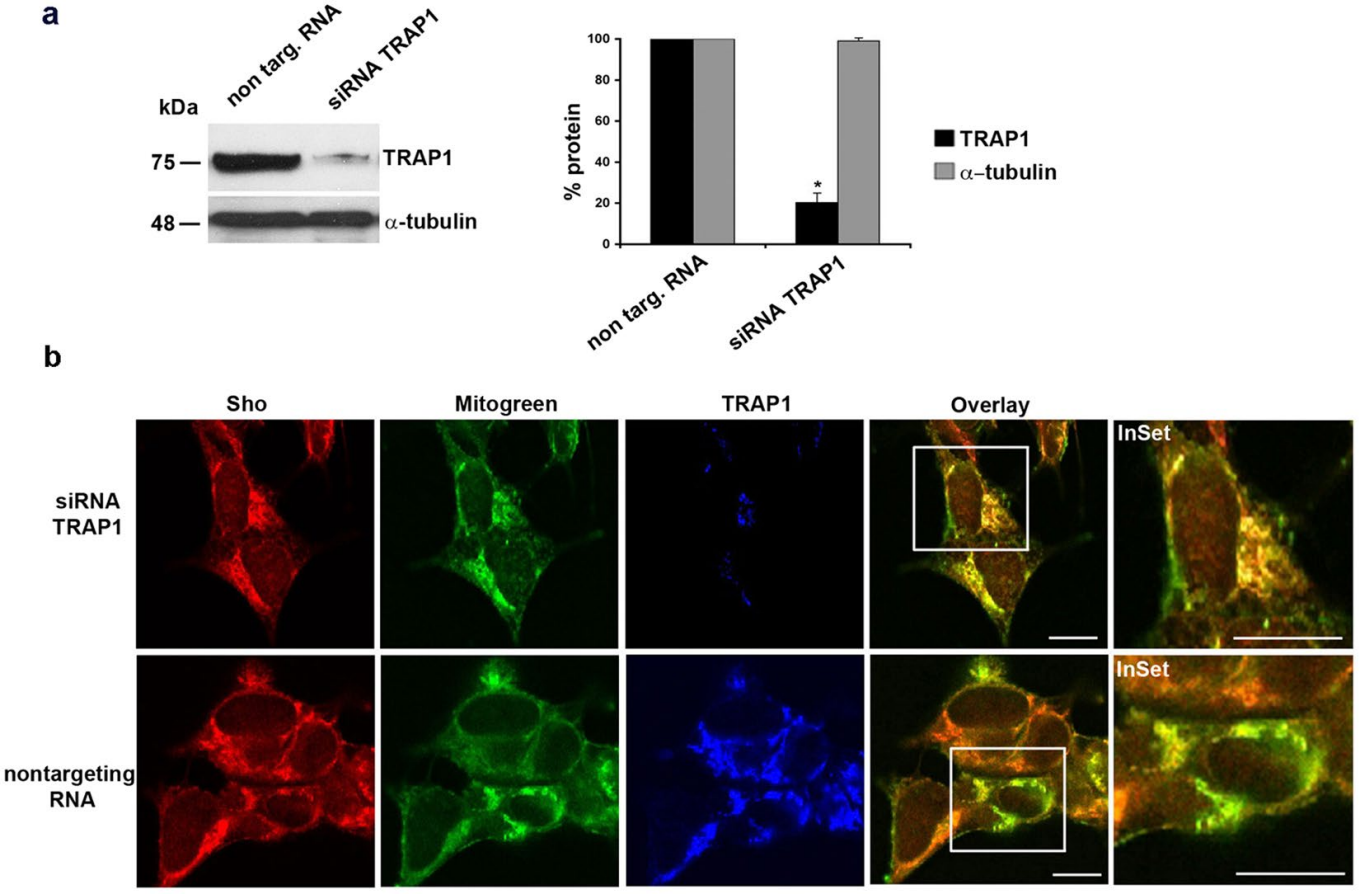
Knockdown of TRAP1 expression regulates mitochondrial localization of Sho in SH-SY5Y cells. (**a**) SH-SY5Y cells were lysed in Buffer 1 and processed for western blotting by using anti-TRAP1 Ab and anti-alpha-tubulin Ab as loading control. TRAP1 was silenced using a specific siRNA for 72 h. In comparison to siRNA TRAP1 cells, 50 μg of total cell lysate from control cells (non targ. RNA) were loaded for reference. PVDF membranes were processed for western blotting analysis by using anti-TRAP1. The same membranes were probed with anti-tubulin Ab followed by ECL detection. The amount of silenced TRAP1 was quantified from three independent experiments. *P < 0.05. (**b**) siRNA TRAP1 SH-SY5Y (upper panel) and non targeting RNA control cells (bottom panel) were processed for triple immunofluorescence analysis by using anti-Sho SPRN-R12 and anti- TRAP1 Ab followed by secondary conjugated Alexafluor-546 and Cy5 Abs, respectively. Mitotracker green (15 min at 37 °C in culture medium) was used to mark mitochondria (green). Overlay is between red and green channels. Colocalization was analysed in at least 52 cells per experiments (N = 3). *P* < 0.03. Scale bars: 10 μm.

To further check for TRAP1 involvement into Sho targeting to mitochondria, TRAP1 expression was knocked-down by using a specific siRNA. The expression levels of TRAP1 were evaluated in control cells (non targeting control siRNA) and TRAP1 silenced SH-SY5Y cells using a specific siRNA for 72 hr. The higher efficiency of the TRAP1-specific siRNA molecule versus a nonspecific control was confirmed by Western blot followed by densitometric analysis (Fig. 4a). Moreover, a comparison of Sho/Mitotracker colocalization signal (see methods for quantification) in TRAP1-knockdown cells (siRNA TRAP1) *versus* control cells (non targeting RNA) showed that the colocalization degree of Sho with mitochondria increased from ~55% in control conditions to ~68% in TRAP1 silenced cells (Fig. 4b). These results were corroborated by a biochemical approach, where we employed the fractionation assay to follow the distribution of the different Sho isoforms in both control cells (shGFP) and TRAP1 knockdown cells (shTRAP1, for 96 h)(see materials for description of the Tet-inducible cell system and Supplementary Fig. S5a). As shown, the 18 kDa Sho isoform specifically enriched the mitochondria under TRAP1 silencing conditions.

Moreover, to dissect where in the cells and which of the Sho isoforms Co-IP with TRAP1, we took advantage of the fractionation assay to perform the Co-IP analysis. As shown in the Supplementary Fig. S5b, the isoforms at 16 kDa and 18 kDa were present in the immunocomplex with TRAP1 only in the microsomal fraction and not in the mitochondrial one. These findings suggest that TRAP1 can control and assist the correct Sho localization at the interface between ER/mitochondrial compartments.

**Figure 5:**
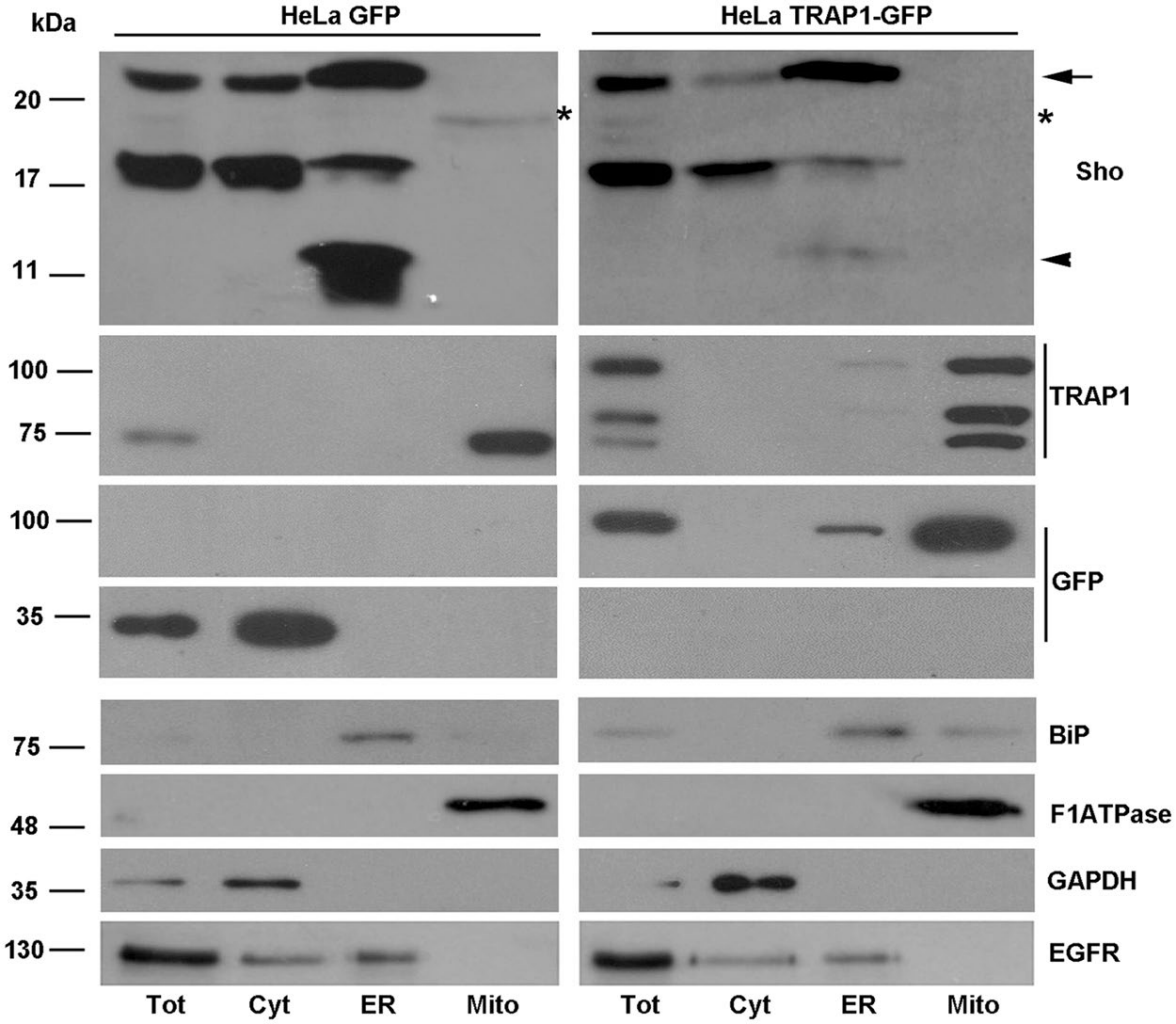
TRAP1 over-expression affects Sho maturation and its targeting to mitochondria. HeLa GFP (control) and HeLa TRAP1-GFP cells (Tetracycline induced TRAP1 over-expressing cells) were subjected to fractionation assay as in Fig. 2. Note the presence of the 18 kDa (asterisk) only in the mitochondria of HeLa GFP cells. The arrow marks the mature isoform and the arrowhead marks the 14 kDa isoform of Sho. The same membranes probed with anti-Sho SPRN-R12 Ab were probed with anti-TRAP1 and anti-GFP Abs. The band between 75 kDa/100 kDa could correspond to cleavage product of the TRAP1-GFP. Anti-BiP, anti-F1ATPase, anti-GAPDH antibodies were used as control of the fractionation assay. The presence of the EGFR (epidermal growth factor receptor) into the cytosolic fraction, could indicate the presence of plasma membrane pieces in the cytosolic fraction.

Consistent with data from Fig. 4, these results support the concept that Sho targeting to ER or mitochondria is effectively dependent on TRAP1 expression. To reinforce this finding, we employed the tetracycline (Tet)-on inducible stable HeLa cell system (Flp-In Trex) expressing either TRAP1-GFP fusion protein or unfused GFP alone as a control. In this cell system, we were able to show by fractionation assay (Fig. 5) that TRAP1 over-expression resulted in the opposite effect observed above, that is the decrement of Sho targeting to mitochondria. These data were strengthened by quantification of immunofluorescence assays where Sho/Mitochondria colocalization was analysed in control (HeLa GFP cells) *versus* TRAP1 over-expressing cells (HeLa TRAP1-GFP induced cells) (Table 1) (see materials for description and Supplementary Figs S6 and S7). Overall, these results indicate that high expression of TRAP1 prevents “aberrant” Sho translocation within mitochondria, most likely by binding it on the endoplasmic reticulum.

**Table 1:** Amount of Sho colocalizing with mitochondria.

HeLa GFP (control)	Sho/Mito	58 ± 0.3%
HeLa TRAP1-GFP (Tet-induced)	Sho/Mito	36 ± 0.5%

Quantification of immunofluorescence assays of Supplementary Fig. S6. Colocalization between Sho and Mitochondria was determined in at least 82 cells from three different experiments under control (HeLa GFP) or TRAP1 induced cells (HeLa TRAP1-GFP). The number of colocalizing red (Sho) and green pixels was normalized for the total number of pixels from an extracted region of interest in the confocal image. The results are expressed in percentage ± S.D; *P* < 0.05.

Moreover, as shown in Fig. 5, since in Tet-induced TRAP1-GFP cells the 22 kDa Sho (arrow) increased with a corresponding decrease of both the immature isoforms at 14 kDa (arrowhead) and 16 kDa (see lane ER, Endoplasmic Reticulum, in the right panel), it is extremely clear that TRAP1 over-expression resulted in an improvement of Sho maturation, possibly increasing translocation of Sho into the ER with a consequent decrement of 18 kDa into mitochondria.

### Acquisition of “prion-like” properties of Sho and lipid rafts association

The role played by TRAP1 in targeting of Sho to mitochondria prompted us to verify its role in Sho folding and whether the mitochondrial Sho was, in some ways, involved in this mechanism. We have previously demonstrated that its paralog protein PrP^C^ was associated with lipid rafts, whose alteration did not affect its cellular distribution, but rather affected its folding^14, 28^, rendering PrP^C^ partially resistant to PK digestion. Besides, it was previously established that Sho bound to anionic lipid surface (by analyses of Sho interaction with cellular membrane models) can assembly into fibrillar structures and that this fibrillization is probably accompanied by the alternation in bilayer structure and the uptake of lipids by the forming fibers^29^.

Based on these observations, we first asked whether Sho was associated with lipid rafts in neuronal cells and if any, which was the role for both lipid rafts and TRAP1 in the folding of Sho.

**Figure 6:**
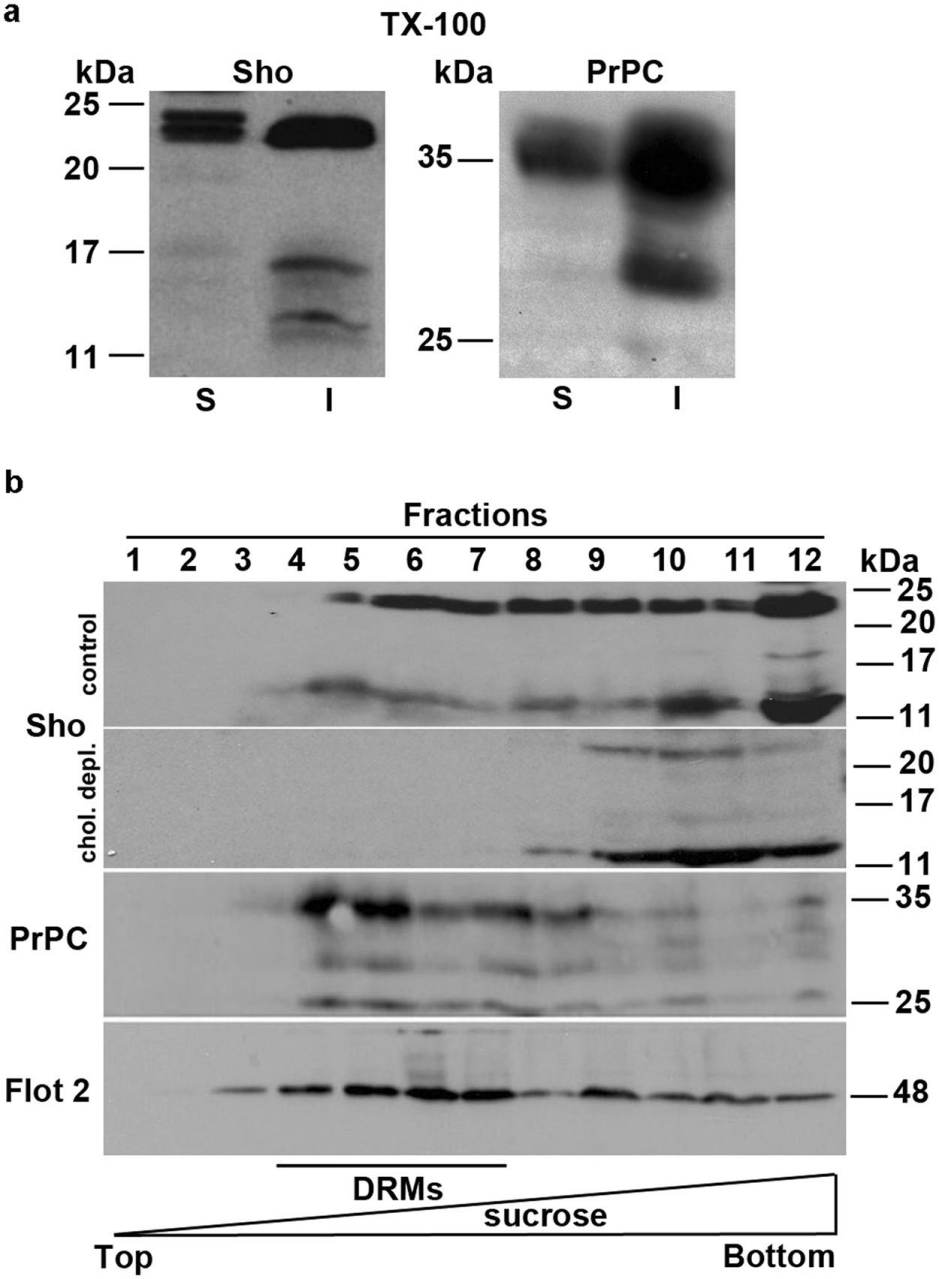
Sho is insoluble in non-ionic TX-100 detergent and is associated to DRMs. (**a**) GT1 cells from 60-mm dishes were washed twice with PBS and then lysed for 20 min on ice in 1 ml of Extraction Buffer. After centrifugation of the lysates, the supernatant (S) represents the TX-100 soluble material. The pellet (I) was then solubilized in 100 μl of solubilization buffer (50 mM Tris pH 8.8, 5 mM EDTA, 1% SDS). Proteins were TCA precipitated from the soluble and insoluble materials and Sho and PrP^C^ were revealed by Western blotting. (**b**) GT1 cells under control or cholesterol depletion conditions, were grown to confluence on 150-mm dishes, washed in PBS and lysed for 20 min in TNE/TX-100 1% (TNE stands for Tris/NaCl/EDTA: 25 mM Tris-HCl pH 7,5, 150 mM NaCl, 5 mM EDTA, 1% TX-100) on ice^13, 21^. A discontinuous sucrose gradient (5–35% TNE, Top-Bottom) was layered on the top of the lysates and one-milliliter fractions (12 fractions in total) were harvested from the top of the gradient. Specifically, starting from the top of the gradient the fractions 4–7 (representing DRMs) and 8–12 (non-DRMs) were collected and loaded on gel. After transfer on PVDF by Western blot, Sho, PrP^C^ and Flotillin-2 (Flot2) were revealed by specific antibodies and ECL. One representative image of three independent experiments is shown.

We found, by TX-100 extraction assay, that Sho, as well as PrP^C^, was in the insoluble (I) material as expected for a DRM-associated protein (Fig. 6a). Interestingly, all Sho isoforms were present in the insoluble fraction. Because TX-100 insolubility can also result from events other than DRM association^30^, we purified TX-100 insoluble microdomains by centrifugation to equilibrium on sucrose density gradients, that allows the segregation of lipid-rich components from the bulk of TX-100 insoluble material^21, 30^. Consistently with the TX-100 extraction assay we found that Sho floated to the DRM enriched fractions (4–7) of the gradient (Fig. 6b), where normally DRM-associated proteins reside (see Flotillin-2 and PrP^C^ ^31^). Notably, both the mature (22 kDa) and the unglycosylated isoform of Sho (~14 kDa) associated with DRMs and, under cholesterol depletion (chol. depl.), almost all of Sho was distributed in the heavier fractions (non DRMs) of the gradient, indicating that DRM-association of Sho was dependent on lipid rafts integrity.

**Figure 7:**
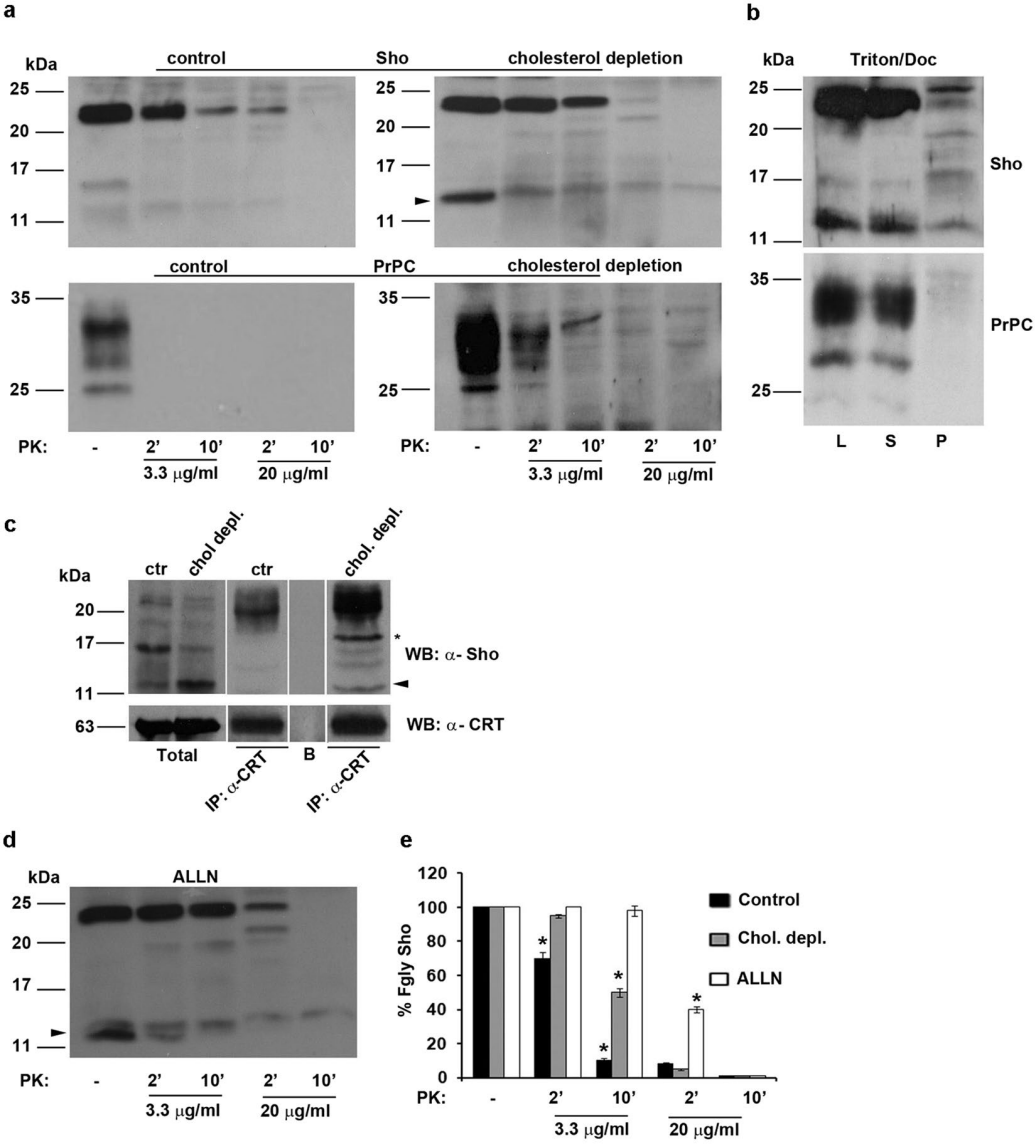
Lipid rafts integrity and proteasomal block regulate Sho folding and its PK- resistance in neuronal cells. (**a**) GT1 cells were grown in 60-mm dishes in control (control) or cholesterol depletion conditions. The cells were lysed in Triton/Doc buffer, in the absence of proteases inhibitors, and where indicated were treated with PK (3.3 μg/ml and 20 μg/ml) for 2 or 10 min or centrifuged (**b**) in a TLA 100.3 rotor at 265,000 *g* and separated into soluble (S) and insoluble (P) materials. In both cases Sho and PrP^C^ were revealed by Western blotting by using SPRN-R12 or SAF32 Ab and ECL. The line L represents 80 μg of total cell lysate before centrifugation. (**c**) GT1 cells were grown as in (**a**) and Calreticulin was immunoprecipitated with anti-CRT primary antibody. The presence of Sho in the immunocomplex was tested by western blotting the IP with anti-Sho SPRN-R12 Ab. To confirm the occurrence of the immunoprecipitate, the membranes were stripped and probed with anti-CRT antibody and ECL. *Indicates 18 kDa Sho; arrowhead indicates the U-gly Sho isoform. Note the presence of both isoforms in the Co-IP with CRT. B: protein-A beads alone. (**d**) The proteasomal pathway is involved in Sho metabolism and in the PK-resistance of Sho. GT1 cells were treated with ALLN (150 μM, 7 hours) and subjected to PK-assay as in (**a**). (**e**) The graph represents percentage of Sho quantified by densitometric analysis relative to PK-assays performed in control, chol. depl. or ALLN treated conditions. The amount of mature Sho (Fgly, fully glycosylated) in not-treated samples (PK -) was considered as 100%. Results were quantified from three different independent experiments and represent the means +/− S.D. *P < 0.05.

Our previous data showing the “protective role” of DRM-association in the correct folding of PrP^C^ ^14^, together with our finding that Sho partitioned in the DRM fractions of the sucrose density gradients, prompted us to investigate the folding of Sho in both GT1 and SH-SY5Y cells, as well as its “prion-like” properties, such as PK-resistance and Triton-Doc insolubility^14, 22, 23^. We reasoned that if Sho possesses a natural tendency to convert to amyloid-like forms *in vitro* ^6^, it should be able to acquire “prion-like” properties also in live cells. To test this hypothesis, we employed two assays that are used to identify the scrapie “prion-like” characteristics of prion protein and its mutants, because they reveal an abnormal folding of the protein. Thus, we first analysed Sho sensitivity to PK digestion under normal conditions, by using PK enzyme on cell lysates of GT1 (Fig. 7a, control) and SH-SY5Y cells (see Supplementary Fig. S8). As expected by our hypothesis, we found that Sho was partially resistant to PK digestion both at 3.3 μg/ml and 20 μg/ml (concentrations at which PrP^C^ was completely sensitive after 2 and 10 min treatment) (Fig. 7a, lower panels and reference^14^). Secondly, differently from cellular prion protein that was present only in the soluble (S) material (see also^14^), about 20% of Sho was recovered in the pellet after ultracentrifugation of Triton/Doc cell lysates (Fig. 7b), indicating that Sho shows a natural tendency to aggregate into the cells.

Overall, these data are consistent with a proteinase-K signature acquisition of Sho under native conditions^6^.

According to our previous findings in epithelial cells^14^, while PrP^C^ was found partially PK-resistant only after 2 min of PK digestion upon cholesterol depletion (Fig. 7a lower panels), Sho was partially PK-resistant already under normal growth conditions in both neuronal cell lines (Fig. 7a, upper panels for GT1 and Supplementary Fig. S8 for SH-SY5Y cells) and its resistance to PK digestion increased of about 30% after cholesterol depletion by methyl-β -cyclodextrin (β CD) (see 10′ at 3.3 μg/ml PK), respect to control. Moreover, after cholesterol depletion the unglycosylated form accumulated (arrowhead), as if lipid rafts could control the maturation of Sho.

### Role of DRM association in Sho folding

To understand in which measure association of Sho with DRMs could be involved in determining the folding of the protein, we sought to check whether in control conditions and after cholesterol depletion, the protein remained associated with the ER chaperones. To test whether and which of Sho isoforms interacts with ER chaperones, we performed Co-immunoprecipitation (Co-IP) assays by immunoprecipitating Calreticulin (CRT) (Fig. 7c), with its specific antibody and revealing Sho by Western blotting with SPRN-R12 antibody. To confirm the occurrence of the immunoprecipitation, Calreticulin was revealed by anti-CRT antibody after stripping of the same membrane. Interestingly, accordingly with its partial resistance to PK digestion under normal growth conditions, we found that mature glycosylated Sho Co-IPed with CRT, which might be indicative of its incorrect folding. Furthermore, upon cholesterol depletion, the amount of mature Sho increased in the immunocomplex with CRT (about 3-fold respect to control) and together with the 18 kDa and unglycosylated isoforms, appeared in the immunocomplex (Fig. 7c).

All these data indicate that Sho possesses a natural tendency to misfold and that the integrity of lipid rafts, as well as for PrP^C^, plays a role in its folding.

### The proteasomal pathway in “prion-like” properties of Sho

It has been proposed that ER retention of misfolded proteins might lead to degradation through the endoplasmic reticulum-associated degradation (ERAD) pathway, as in the case of some pathological PrP mutants^15, 32, 33^. Thus, we checked for the role of this pathway in the “prion-like” properties of Sho.

GT1 cells were treated with the proteasome inhibitor ALLN (150 μM for 7 h) and subjected to PK-assay. In agreement with Pfeiffer *et al*.^25^, we found that under proteasomal block the unglycosylated Sho form accumulated into the cells (Fig. 7d, PK-). Second, by comparing the bands of PK digestion under proteasomal block (ALLN) to control conditions (Fig. 7a, upper panel control), both mature and immature 14 kDa Sho, resulted almost completely resistant to PK treatment, both after 2′ and 10′ at 3.3 μg/ml (Fig. 7d, ALLN and compare with control 7a, upper panel). While in control conditions after 10′ at 20 μg/ml of PK, Sho resulted almost completely digested, we calculated that after ALLN treatment (Fig. 7e), about 40% of fully glycosylated Sho resulted resistant to PK treatment and that the unglycosylated isoform accumulated and was partially PK resistant.

### TRAP1 over-expression and Sho PK-resistance

**Figure 8:**
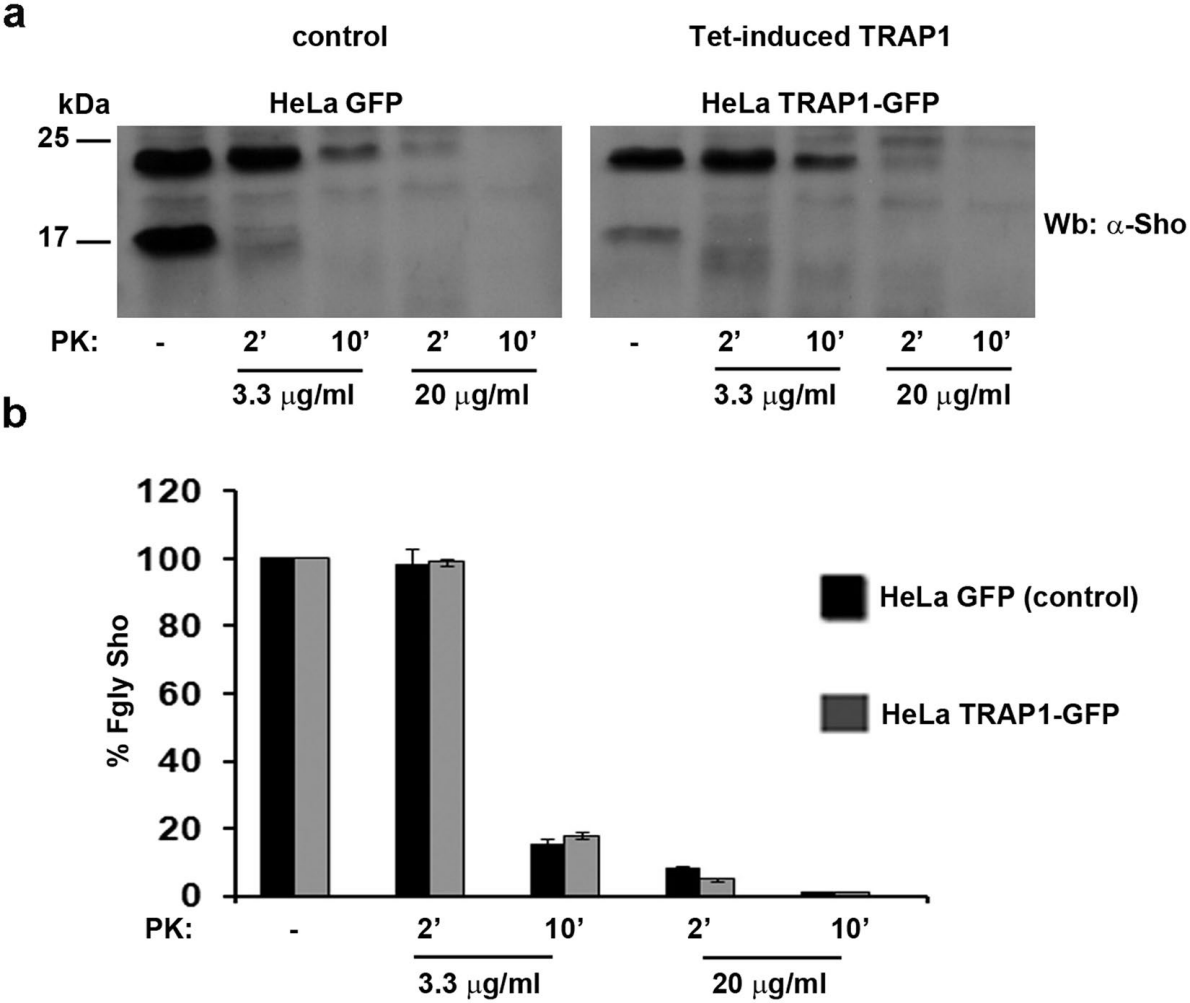
TRAP1 over-expression does not affect PK-resistance of Sho. (**a**) Both HeLa GFP (control) and HeLa TRAP1-GFP (over-expressing TRAP1) cells were subjected to PK assay as in Fig. 7. Note that TRAP1 over-expression does not affect PK-resistance of Sho but influences the immature/mature Sho ratio (see right panel TRAP1-GFP cells, lane PK- and compare with HeLa GFP control). (**b**) Quantification of Fgly Sho PK-resistance was performed as in Fig. 7.

To investigate the role of TRAP1 in Sho PK-resistance, we used the PK-assay in the tetracycline-induced HeLa TRAP1-GFP expression system (see materials for description). As shown in the Fig. 8, in control cells (HeLa GFP), as well as in GT1 cells (see Fig. 7), Sho resulted partially PK-resistant after 2′ and 10′ of PK treatment. The same result was found in TRAP1 over-expressing cells, where, as indicated in the fractionation assay in previous Fig. 5, the most represented Sho isoform was the mature one (22 kDa), reinforcing the data by which TRAP1 over-expression results in an improved ER translocation of Sho and consequently in its increased maturation. However, although TRAP1 over-expression seems to not affect the natural PK-resistance of the mature Sho isoform (Fgly), we cannot exclude that TRAP1 expression levels could impact on the folded/unfolded Sho isoforms ratio.

## Discussion

A depletion in Sho has been demonstrated in several experimental scrapie-infected rodents and in naturally infected scrapie sheeps^34–36^. The similarity between N-terminals of Sho and PrP^C^, together with demonstration of their interaction by surface plasmon resonance and yeast two-hybrid analysis^37^, suggests that the two proteins share biological functions. Thus, a functional link between mammalian PrP and Sho and a possible involvement of Sho in conformational transition of PrP^C^ to its misfolded scrapie isoform PrP^Sc^ has been postulated.

In line with a previous report^38^, we found that Sho was localized on the plasma membrane. Attached to the cell surface, Sho would be positioned to act as a receptor for ligands found at the cell surface^39^ and may have a role in cell signaling, similar to PrP which binds the neural cell adhesion molecule participating in the tyrosine kinase fyn signaling pathway leading to neurite outgrowth^40^.

The recently proposed channel activity of Sho^41^, which is also characteristic to some pathogenic PrP mutants, may be linked to a physiological function of Sho.

We found that Sho was expressed as two major bands likely corresponding to the unglycosylated and glycosylated isoforms (Fig. 1) and that it was partially localized in the ER and mitochondria of both neuronal GT1 and SH-SY5Y, as well as of non-neuronal HeLa cells.

Based on previous evidence that the pathogenic effects of PrP^Sc^ could be related to the presence of misfolded forms in the ER^15, 42^ and that the ER localization and DRM association of PrP^C^ in the early secretory pathway was crucial for its correct folding^14^, the partial ER localization of Sho could have an impact on its folding properties. Sho shows a highly conserved N-terminal signal sequence; Arg-rich basic region containing up to six tetrarepeats of consensus XXRG (where X is G, A or S)^4^ and the overall structure appears closely related to prion protein. These motifs can have a role in the packaging of amyloid fibers^43^. However, the bulk of the homology between Shadoo and PrP^C^ is found within the hydrophobic tract, which is one of the most conserved regions amongst PrP sequences from diverse organisms, perhaps implying functional homology^44^.

In concordance with its homology to the unstructured N-terminus of PrP^C^, Shadoo, as well as prionogenic proteins^44, 45^, possesses a very high level of intrinsic disorder^4^.

Interestingly, we found that a 18 kDa form of Sho was targeted to mitochondria and together with the 16 kDa interacted with the molecular chaperone TRAP1, exclusively in the ER fraction and not in the mitochondria. In agreement with a role for TRAP1 outside mitochondria, for quality control of mitochondrial proteins^46^, our findings prompted us to hypothesize that TRAP1 could possibly act as a “controller” of the correct Sho localization at the interface between the ER/mitochondrial compartments.

Mitochondrial localization of Sho was in agreement with a recent description that the ER signal peptide of Sho, as well as of amyloid precursor protein (APP), has the property to mediate alternative targeting to mitochondria^24^, and that the dual targeting to either the ER or mitochondria is mediated by structural features (alfa-helical domains and/or GPI signal sequence) within the nascent chain. Moreover, based upon molecular weight prevision, the 18 kDa Sho could correspond to Sho molecules bearing an intact signal peptide, as previously shown for PrP^C^ ^15, 47^. However, further experiments are needed to show this assumption.

Whether TRAP1 could function to assist and/or mediate Sho mitochondrial localization was assessed, in this study, by both a specific siRNA approach and over-expression system. Indeed, while knockdown of TRAP1 expression led to a preferential targeting of Sho to mitochondria, TRAP1 over-expression exerted the opposite effect, strongly decreasing Sho mitochondrial accumulation, thus defining a crucial role for TRAP1 in the regulation of the dual Sho targeting.

Moreover, the acquisition of the PK-resistance of Sho occurs despite the amount of translocated Sho. As previously proposed also for APP protein^25^, the mistargeting of secretory proteins to cytosol or mitochondria may challenge protein homeostasis and causes toxicity, contributing to pathomechanisms. Further studies are needed to elucidate this issue.

Interestingly, we found that the 18 kDa Sho isoform associates to Calreticulin under cholesterol depletion, indicating its misfolding and that Sho has a natural tendency to aggregate. Thus, the question arises as for why Sho becomes misfolded in normal growth conditions.

It has been proposed that the folding of newly synthesized polypeptide chains into their native conformations, and the unfolding of proteins from their native states, proceeds through distinct intermediates from which derive non-native oligomeric species of different sizes and structures which are able to self-associate to form non-native oligomeric species of different sizes and structures^48^. As the polypeptides involved in Prion, Parkinson’s, Alzheimer’s and Huntington’s diseases populate a wide variety of folding intermediates, they have a higher propensity to form such oligomeric species^49^. On the basis of previous works where Sho was described to be the product of a prion-related gene and to represent a prion member family^4^, and that Sho was found able to aggregate and form amyloid structures resembling that of PrP^6^, we can speak about prion protein-like properties meaning that being Sho a prion-related protein it could be able to misfold and aggregate as prions do. Thus, analyzing our results, we propose that Sho could be a new member of this protein family and that the presence of its intermediate isoforms could regulate its folding properties. In this context, overall our findings support the network model for prion-like proteins^45^.

However, our results are in line with recent data showing that recombinant mouse or sheep Sho converted to an aggregated/amyloid form without recourse to chemical modification (denaturation or acidification, etc.), thus defining a proteinase-K signature for Sho under native conditions^6^. In addition, it was interesting to note that under cholesterol depletion, with respect to control conditions, the unglycosylated Sho accumulated indicating that lipid rafts contribute to Sho folding. Consistently, the fact that under cholesterol depletion the presence of mature Sho increased in the immunocomplex with CRT together with the 14 kDa one, suggests that integrity of lipid rafts has a role in the maturation/processing of Sho and the presence of Sho isoforms in the pellet of the Triton/Doc assay, reinforces the idea that they were aggregated.

Strikingly, as clearly shown in Fig. 7, Sho was found partially resistant to PK digestion under control conditions and its resistance was reinforced by cholesterol depletion. These results are in contrast with the behaviour of PrP^C^, which in control conditions is completely digested by PK, while they are in agreement with the fact that PrP^C^ becomes partially misfolded and PK-resistant after cholesterol depletion.

However, it is known that proteins which do not pass the quality control system, are retained in the ER and retrotranslocated into the cytosol to be then degraded by the proteasome^50^. Interestingly, the ERAD pathway was described to be related to the cytotoxicity of PrP^51^ and to some PrP pathological mutants^52^. Moreover, in agreement with very recent findings which describe, in both cultured cells and in scrapie-infected rodents, disruption of glycosylation and enhancement of Sho proteasomal degradation^53^, we found that the proteasomal inhibitor ALLN led to the accumulation of immature unglycosylated band of Sho (see also ref. 25).

While confirming the involvement of proteasomal pathway in the degradation of unglycosylated Sho isoform, we asked why proteasomal block induced a PK-resistance of Sho.

One explanation comes from analysis of the Co-IP assays between Sho and ER chaperones (Fig. 7c) showing that, in normal condition, mature 22 kDa Sho was present in the immunocomplex with the ER chaperone Calreticulin (previously suggested by Watts *et al*.)^54^, strongly indicating that Sho was tendentially misfolded. Furthermore, our finding that under cholesterol depletion, not only translocated Sho intermediate forms Co-IPed with CRT, but also the 18 kDa form (Fig. 7c), could be explained by the existence of cytosolic CRT which can be generated by an inefficient ER import^55, 56^.

We can conclude that Sho is associated to DRMs and acquires “prion-like” properties in both neuronal and non-neuronal cells. These features fit well to protein that belongs to the family of naturally disordered and prionogenic proteins^44^. Moreover, cholesterol depletion leads to accumulation of its immature unglycosylated form and, while the unglycosylated Sho was degraded *via* the proteasome, block of the latter accounts for the accumulation of this form into the cells and to the increase of Sho misfolding confirmed by the increase of its PK-resistance. Moreover, our data for the first time underlie the crucial role of the mitochondrial chaperone TRAP1 in the dual ER/Mitochondrial targeting of Sho.

In the light of a very recent finding^16^ describing a key role for Sho in the folding pathway of PrP, it remains to be further investigated which is the impact of misfolded Sho in the metabolism of PrP^C^ and/or its pathological mutants.

## Electronic supplementary material

Supplementary information
