# Supplementary material for: Regulation of sub-compartmental targeting and folding properties of the Prion-like protein Shadoo
Source: Sci Rep. 2017 Jun 16;7:3731. doi: 10.1038/s41598-017-03969-2 (PMC5473912; doi:10.1038/s41598-017-03969-2)
Supplement: Supplementary file 1 — Supplementary information [file 41598_2017_3969_MOESM1_ESM.pdf]

Supplementary Information

Regulation of sub-compartmental targeting and folding properties of Prion-like protein Shadoo

Anna Pepe [1], Rosario Avolio [1], Danilo Swann Matassa [1], Franca Esposito [1], Lucio Nitsch [1], Chiara Zurzolo [1,3], Simona Paladino [1,2] and Daniela Sarnataro [1,2,*]

[1]Department of Molecular Medicine and Medical Biotechnology, University of Naples "Federico II", Via Pansini 5-80131, Naples-Italy; [2]Ceinge-Biotecnologie avanzate, s.c.a r.l., Via G. Salvatore 486-80145, Naples-Italy; [3]Unité de Trafic Membranaire et Pathogenese, Institut Pasteur, 25-28 Rue du Docteur Roux, 75724 Paris CEDEX 15, France.

Figure S1. Sho is N-glycosylated in SH-SY5Y cells. The cells were grown on dishes and Peptide N-glycosidase (PGNaseF) digestion (5 units/sample) was performed on 1mg of cell lysates. Treated (+) or not (-) samples were loaded on gels and then analyzed by SDS-PAGE and Western blotting with anti-Sho Ab. F-gly: fully glycosylated, U-gly, unglycosylated. The band of Sho at ~16kDa is sensitive to PNGaseF and could be an intermediate glycosylation product.

Figure S2. Sho is localized both in the ER and to the cytoplasmic surface of the ER membrane. SH-SY5Y cells were grown on coverslips and treated with cold digitonin before fixing with 2% paraformaldehyde and eventually permeabilized with 0.075% saponin (+). The cells were then incubated with either anti-Sho Ab (SPRN-R12) or with antibodies against CNX (marker of ER membrane) or PDI (lumen ER protein). PCC= 0.66 for Sho/CNX; PCC=0.72 for Sho/PDI. $P<0.05$. Scale bars: 10 μm.

Figure S3. Sho is localized in mitochondria. SH-SY5Y cells were grown on coverslips and

treated as in Fig. S2, with the exception that here the cells were incubated with both anti-Sho Ab (SPRN-R12) and with antibodies against TOM20 (outer mitochondrial membrane) or F1ATPase (inner mitochondrial membrane), followed by secondary antibodies conjugated to Alexafluor-546 or -488. PCC=0.12 for Sho/TOM20; PCC=0.82 for Sho/F1ATPase. $P<0.05$. Scale bars: 10 μm.

Figure S4. Sho is localized on the plasma membrane and does not accumulate in the Golgi apparatus. a) GT1 cells were grown as in Figure 1b and processed for indirect immunofluorescence analysis by double immunolabelling with the SPRN-R12 anti-Sho Ab and anti-Golgb1 (upper panel) or anti-PrP Ab (lower panel) followed by secondary Abs conjugated with Alexafluor-546 and Alexafluor-488, respectively. Nuclei were stained with DAPI dye. Arrows point to cell surface. Scale bar: 10 μm. **b)** GT1 cells were biotinylated at cell surface and biotinylated proteins were immunoprecipitated with streptavidin beads (see methods). Sho was revealed by SPRN-R12 Ab after SDS-PAGE, western blotting and ECL. Tot: total cell lysate; IP: streptavidin beads and represents biotinylated Sho; SN: supernatant of the IP.

Figure S5. A major amount of 18kDa Sho is targeted to mitochondria under TRAP1 knockdown and with the 16kDa coimmunoprecipitates with TRAP1 in the microsomal fraction. a) Control (ctr, shGFP) and TRAP1-silenced HeLa cells (shTRAP1) were processed for fractionation assay as in Figure 5. The presence of 18kDa (asterisk) increases in the mitocondrial fraction of shTRAP1 cells. F1ATPase, BiP and GAPDH have been used as control of the procedure. **b**) Control (ctr, shGFP) and TRAP1-silenced HeLa cells (shTRAP1) were processed as in **a**) with the exception that here TRAP1 was immunoprecipitated from each fraction and Sho was revealed by anti-Sho Ab. Asterisk indicates 18kDa Sho and arrow points to 16kDa isoform. In the Input, BiP and F1ATPase were used as control of the correct fractionation (bottom panels).

Figure S6. TRAP1 over-expression affects mitochondrial targeting of Sho. HeLa GFP and HeLa Tet-TRAP1-GFP cells were treated as in Figure 4b, with the exception that here, after fixation, the cells were immunolabelled with anti-Sho SPRN-R12 Ab and anti-TRAP1 Ab followed

by secondary conjugated Alexa-546 and Cy5 Abs, respectively (upper panel). Note: for homology with the panel below, the TRAP1 signal was acquired with a 633nm laser but was visualized in green. HeLa TRAP1-GFP Tet-induced cells were immunostained only with anti-Sho SPRN-R12 Ab followed by secondary Alexa-546 conjugated Ab to visualize Sho (red). TRAP1 was visualized (green) thanks to GFP fluorescence signal. Colocalization between Sho and Mitochondria was determined as described in Table 1. Scale bar: 10 μm.

Figure S7. TRAP-1 over-expression does not abolish Sho targeting to mitochondria. This figure represents a more exposed gel of right panel Figure 5. Note that in HeLa TRAP1-GFP Tet induced cells the 18kDa Sho (*) is present in the mitochondrial fraction, even if in reduced amount respect to control condition (see Figure 5).

Figure S8. Sho is partially PK-resistant also in human neuronal SH-SY5Y cells. SH-SY5Y cells in control or cholesterol depletion conditions, were processed for PK assay. The cells were grown as in Figure 7 (see also materials and methods in the main manuscript) and subjected to PK treatment for 2’ or 10’ with 3.3μg/ml of enzyme. Sho was revealed with anti SPRN-R12 Ab by Western blotting and ECL. Note the accumulation of 14kDa Sho in chol. depl. cells and the PK-resistance of both the mature and the immature Sho isoforms.

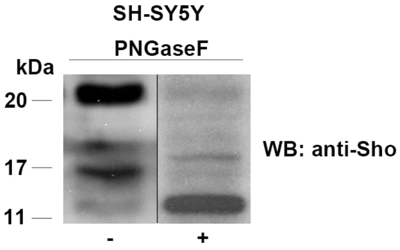
SH-SY5Y
PNGaseF
kDa
20
17
11
-
+
WB: anti-Sho

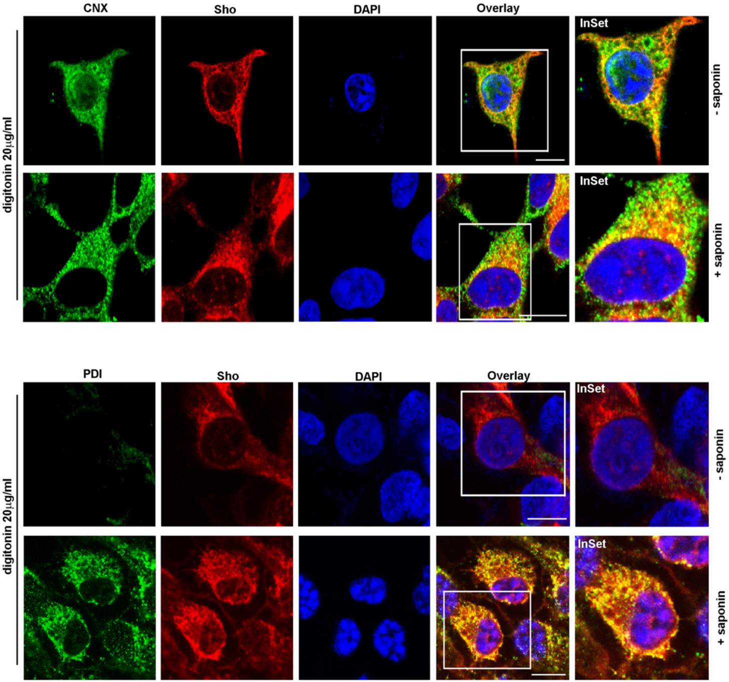
CNX
Sho
DAPI
Overlay
InSet
- saponin
InSet
+ saponin
digitonin 20μg/ml
PDI
Sho
DAPI
Overlay
InSet
- saponin
InSet
+ saponin
digitonin 20μg/ml

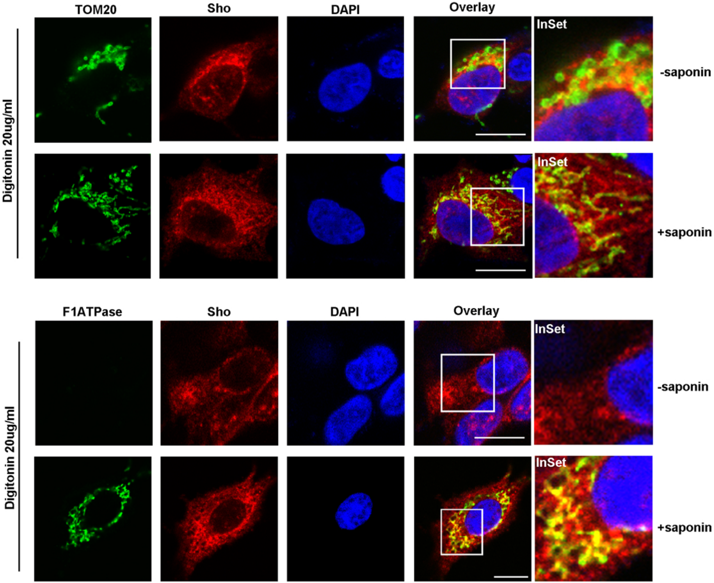
TOM20
Sho
DAPI
Overlay
InSet
-saponin
InSet
+saponin
Digitonin 20ug/ml
F1ATPase
Sho
DAPI
Overlay
InSet
-saponin
InSet
+saponin
Digitonin 20ug/ml

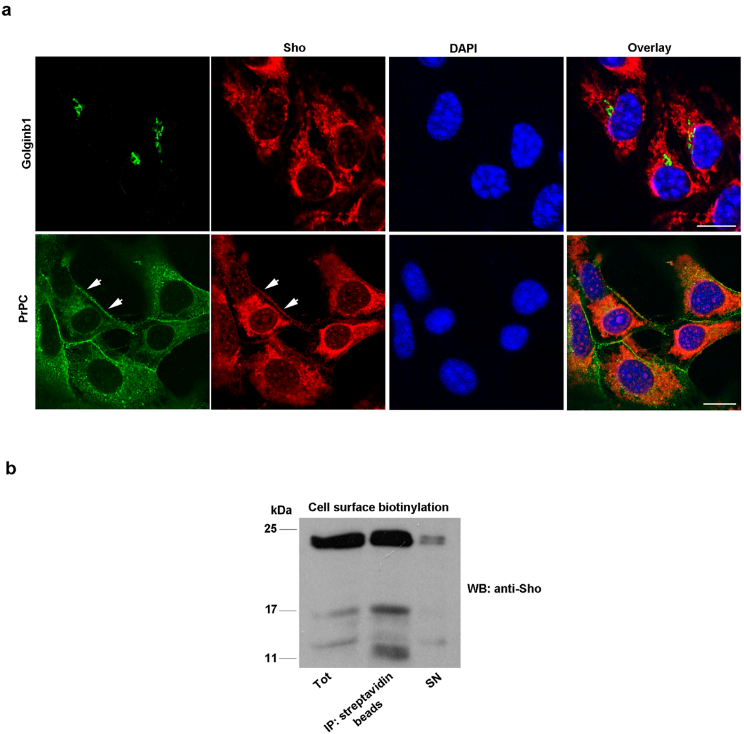
a
Sho
DAPI
Overlay
Golginb1
PrPC
b
kDa
Cell surface biotinylation
25
17
11
WB: anti-Sho
Tot
IP: streptavidin beads
SN

a

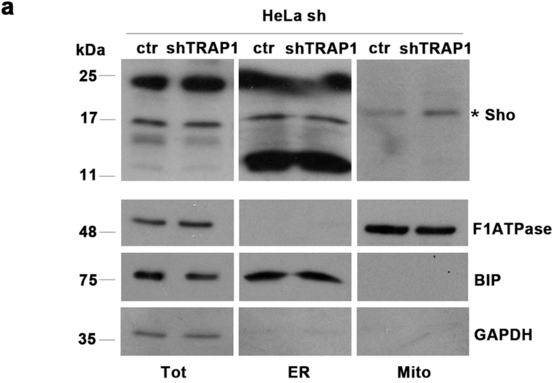
HeLa sh
kDa
ctr
shTRAP1
ctr
shTRAP1
ctr
shTRAP1
25
17
11
* Sho
48
F1ATPase
75
BIP
35
GAPDH
Tot
ER
Mito

b

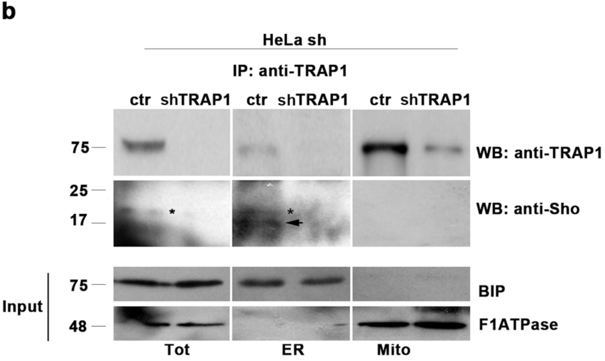
HeLa sh
IP: anti-TRAP1
ctr
shTRAP1
ctr
shTRAP1
ctr
shTRAP1
75
WB: anti-TRAP1
25
17
*
*
WB: anti-Sho
Input
75
BIP
48
F1ATPase
Tot
ER
Mito

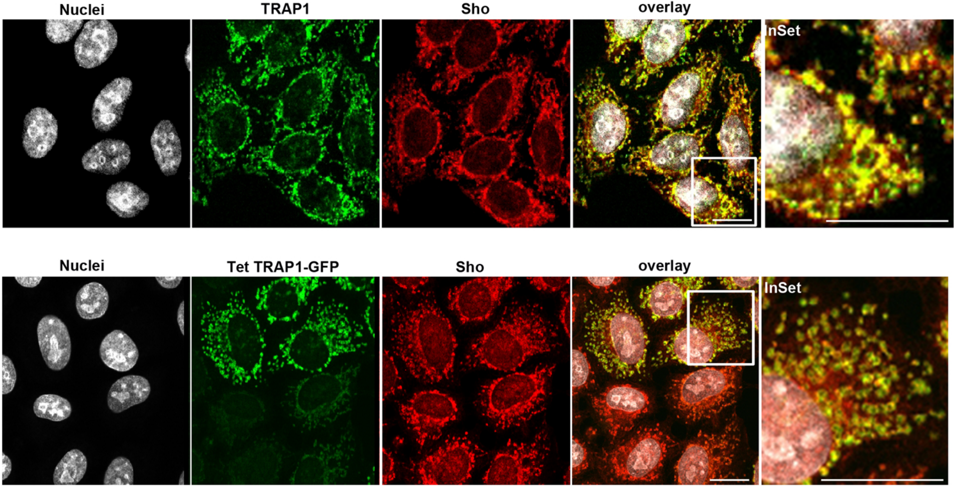

Nuclei
TRAP1
Sho
overlay
InSet
Nuclei
Tet TRAP1-GFP
Sho
overlay
InSet

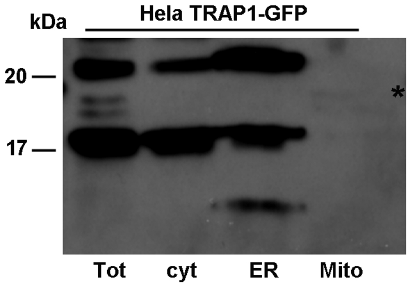
kDa
Hela TRAP1-GFP
20
17
*
Tot
cyt
ER
Mito

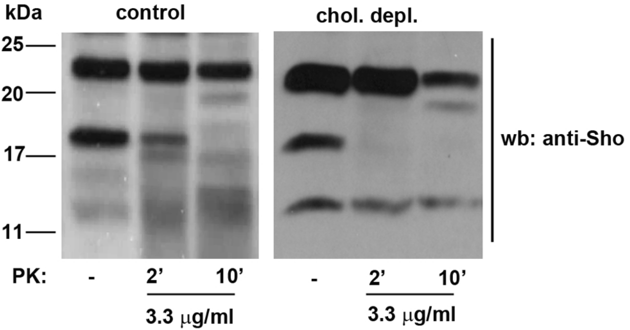

kDa
control
chol. depl.
25
20
17
11
wb: anti-Sho
PK:
-
2'
10'
-
2'
10'
3.3 μg/ml
3.3 μg/ml
